# In the moment, out of reach? Experience sampling with adolescents in the context of school smartphone bans and shifting societal norms

**DOI:** 10.1111/jora.70118

**Published:** 2026-01-08

**Authors:** Michelle Achterberg

**Affiliations:** ^1^ Department of Psychology, Education and Child Studies (DPECS) Erasmus School of Social and Behavioral Sciences (ESSB), Erasmus University Rotterdam (EUR) Rotterdam The Netherlands

**Keywords:** adolescents, compliance, ecological momentary assessment, engagement, experience sampling method, methodology, smartphone restrictions

## Abstract

Experience sampling method (ESM) research, relying on real‐time data collection via mobile devices, provides unique insights into adolescents' daily lives. However, concerns about digital distraction and overstimulation have led to shifting societal norms and consequently, increased restrictions on smartphone use—both institutionally (e.g., school bans) and informally (e.g., parental rules, self‐regulation). These constraints raise questions about the feasibility and ecological validity of using ESM in adolescent samples. In this study, 195 adolescents (*M*_age_ = 16.12) participated in a 17‐day ESM protocol, completing six prompts daily. Most adolescents reported facing school‐based (88%) and parental (56%) smartphone restrictions. Despite these constraints, compliance was moderate to high (*M* = 78%), and analyses of nonresponse patterns revealed when and why prompts were most likely to be missed. Early morning prompts were often missed due to sleep, late morning prompts due to school, and evening prompts due to work—highlighting the importance of context‐sensitive sampling strategies. Moreover, data quality was high: careless responding was rare, and participants reported high levels of integrity and motivation. Most participants evaluated the study as positive, with financial incentives, scientific contribution, and social connection as key motivators. These findings underscore that adolescent ESM studies remain feasible and ecologically valid when protocols are flexibly aligned with real‐world constraints. Given that societal norms on digital well‐being are in flux and smartphone restrictions intensify, aligning ESM design with adolescents' everyday realities becomes increasingly essential to preserve both feasibility and ecological validity in research on adolescence.

## INTRODUCTION

### The digital world in flux: Notifications, norms, and new restrictions

In recent years, experience sampling method (ESM), also known as ecological momentary assessment (EMA, Stone & Shiffman, 1994), has become an increasingly popular approach to study the everyday experiences and emotional lives of adolescents. ESM allows researchers to capture moment‐to‐moment fluctuations in affect, behavior, and context, by prompting participants multiple times per day via mobile devices (Myin‐Germeys et al., 2018). Especially during adolescence—a developmental period marked by heightened emotional reactivity and increased sensitivity to social contexts (Blakemore, 2008; Mulder et al., 2024)—ESM offers a powerful lens into real‐time processes that are difficult to capture through retrospective or cross‐sectional methods (Silk et al., 2003; Stone & Shiffman, 1994; van Roekel et al., 2019).

However, the societal and technological landscape in which ESM operates is shifting rapidly: prompts (i.e., notifications to fill out questionnaires) are now easily lost among hundreds of daily smartphone notifications. Recent survey data indicate that adolescents receive an average of 237 notifications per day, all in competition for adolescents' attention (Radesky et al., 2023). Moreover, across several countries, concerns about adolescent screen time, distraction, and overstimulation have prompted policy changes—most notably, school‐wide smartphone bans during schooltime (Brummer & Achterberg, 2025; Magnusson et al., 2023). These regulations, introduced to reduce digital overload, improve concentration and academic success, and support social safety and mental well‐being, are not limited to schools. Many adolescents also face increasing restrictions at home: some are required to hand in their smartphones during dinner or at night, others voluntarily switch them off to reduce distractions or have a complete “Digital Detox” (Salepaki et al., 2025). Such practices, driven by shifting societal norms around digital hygiene and well‐being (Vanden Abeele, 2020), limit adolescents' availability for ESM studies during key parts of the day.

While these shifts may benefit adolescent well‐being, they introduce critical methodological challenges for ESM research. One of the main strengths of ESM lies in its ecological validity—capturing experiences as they naturally unfold in daily life (Scollon et al., 2003; Stone & Shiffman, 1994). Yet as adolescents become less reachable across specific time windows, researchers must reconsider how, when, and through which channels prompts are delivered. This raises an increasingly pressing question: is it still feasible to conduct in‐the‐moment ESM studies with adolescents?

### Methodological tensions: Reachability versus realism

To date, limited empirical work has explored how ESM designs can respond to changing societal norms and institutional practices related to smartphone availability. One advanced feasibility study involving early adolescents used a custom‐built app to track daily experiences, achieving high compliance by aligning prompts with daily transitions to and from school (Chin et al., 2016). Other work suggests that protocols synchronized with institutional rhythms and supported by school staff may enhance compliance (van Roekel et al., 2019). Although ESM studies have always faced constraints due to contextual and logistical factors, the recent implementation of school‐wide smartphone bans has significantly intensified these challenges. In several countries, including the Netherlands, national school smartphone bans have now been implemented (Brummer & Achterberg, 2025), further complicating the feasibility of in‐the‐moment data collection during school hours. While aligning ESM protocols with school‐free hours may improve feasibility (Bülow et al., 2022), it risks omitting key aspects of adolescents' daily experience, particularly those tied to academic stress, peer dynamics, and social interactions. Capturing only pre‐ and post‐school data may therefore yield a biased picture of adolescents' social and emotional functioning, undermining the ecological validity of studies focused on performance pressure, peer conflict, or classroom‐related affect. These trade‐offs highlight the need for nuanced study designs that balance feasibility with ecological validity, particularly in research targeting adolescence.

Thus, as smartphone access becomes increasingly regulated—both formally through school policies and informally through parental rules or adolescent self‐regulation—there is an urgent need to understand how such constraints affect the feasibility and ecological validity of in‐the‐moment data collection. The present report aims to contribute to this discussion by evaluating the feasibility of an ESM protocol among adolescents (14–17 years), situated within the context of smartphone regulations at school. A total of 195 participants completed six daily prompts over 17 days using the Avicenna Research app. The Avicenna Research app is a secure platform designed for ecological momentary assessment that supports customizable scheduling, offline functionality, and GDPR‐compliant data storage. We selected this app given its robustness for intensive sampling and prior successful use in adolescent ESM studies within our research groups (Luijk et al., 2023; Van der Kaap‐Deeder et al., 2023). To accommodate limited device access, prompts were carefully scheduled around school transitions and lunch breaks, with wide response windows. Moreover, teachers and parents were provided with explanatory materials to encourage institutional and parental support. This report describes the design decisions, logistical adaptations, and practical lessons learned from this study. It reflects on the broader implications for ESM, on how changes in societal norms concerning digital technology use may impact the future of ambulatory psychological assessment in research on adolescents.

## METHODS

### The case study

The study was part of the Leiden Consortium on Individual Development (L‐CID; Crone et al., 2020). For detailed information about the study design and recruitment procedures, see Achterberg et al. (2018) and Crone et al. (2020). At wave 8, all families who had previously expressed interest in continued participation were recontacted via paper postcard (*N* = 300 adolescents; see OSF metadata for materials). We deliberately chose to send a physical postcard via traditional mail, as such a tangible message might be more noticeable than digital messages, which adolescents may easily overlook amid the constant stream of digital notifications and emails that adolescents typically receive (Radesky et al., 2023). Interested adolescents could register via a QR code and indicate their preferred mode of communication. In total, 211 adolescents signed up (70%); after attrition, 195 adolescents (*M*_age_ = 16.12, SD = 0.79; 52% female) completed the full ESM protocol. Participants came from various schools across the Netherlands, and 85% of the participants lived in the West of the Netherlands (South Holland, North Holland, Utrecht). Ninety‐nine percent of participants identified as ethnically Dutch, whereas 7% reported having multiple ethnic backgrounds (e.g., Moroccan, Surinamese, Indonesian). The sample was therefore highly homogeneous in terms of race/ethnicity. Regarding socioeconomic status, 68% of the participants had at least one parent who completed higher education (comparable to a bachelor's degree). Participants were enrolled in secondary education in the Netherlands, which is divided into different academic tracks: prevocational (VMBO/MAVO), general secondary (HAVO), or preuniversity (VWO, including gymnasium). Informed consent was obtained digitally via Qualtrics from all participants, and if necessary (i.e., participants <16), from legal guardians. Participants received €7.50 for completing the start questionnaire and an additional €2.50 for each day they completed prompts during the 17‐day ESM period, allowing them to earn up to €50 in total. During the study, information for the adolescents (designed to be accessible and engaging through the use of visuals and emoticons) was sent throughout the most preferred mode, WhatsApp. Parents received a separate information letter via email. In addition, both adolescents and parents were provided with a school‐oriented information letter that could be forwarded to school staff. All study materials are available via OSF metadata.

### Nationwide school smartphone ban

In January 2024, the Netherlands introduced a national policy restricting smartphone use during secondary school lessons. Although the policy states that phones should not be used during instructional time unless explicitly required for educational purposes, schools retain substantial autonomy in how the regulation is implemented and enforced. As documented in our public report (Brummer & Achterberg, 2025) and a concurrent preregistered mixed methods study (Brummer et al., 2025), this results in considerable variability across schools. This heterogeneity was also reflected in our sample: based on open‐ended questionnaire responses, adolescents reported policies ranging from no explicit rules (11%), “phone out of sight” during lessons (16%), hand‐in procedures using phone pouches (24%), phone use allowed only during breaks (12%) to full‐day prohibitions requiring phones to remain in lockers (35%). Thus, although all adolescents in this study were affected by the national ban, the practical experience of the ban differed across participants. Further qualitative detail on how these policies are enacted in practice can be found in Brummer et al. (2025).

### The sampling strategy

To navigate constraints such as limited smartphone access during school hours and digital overload, the sampling strategy was deliberately adapted to maximize both feasibility and in‐the‐moment data quality. In designing the sampling strategy for this study, established guidelines for ESM research were followed (Myin‐Germeys & Kuppens, 2022). Given the nationwide school smartphone ban that came into effect during the setup phase of this study, we deliberately implemented a 17‐day protocol, including both 11 school days and six weekend days. Participants received six prompts per day via the Avicenna app (see Table 1), yielding a maximal total of *t* = 102 observations per person. Even if school‐time prompts were consistently missed, participants could still provide up to 80 observations—sufficient for within‐person analyses (Revol et al., 2024). Importantly, prompts were scheduled around school breaks and class transitions to maximize the chances of in‐the‐moment responding despite restricted phone access during lessons. In the Netherlands, secondary school classes typically last 50 min. We therefore applied a 60‐min response window, ensuring that prompts could be answered between lessons. In addition, most schools have a lunch break around noon, which was incorporated into the sampling scheme to maximize feasibility. This approach is supported by prior research emphasizing the importance of aligning prompt timing with adolescents' natural routines, especially within institutional settings (Chin et al., 2016; van Roekel et al., 2019).

**TABLE 1:** Sampling scheme.

No.	Moment	Prompt time^a^	Considerations
1	Early morning	7:55–8:10	Possibly not yet awake
2	Morning	11:30–12:00	Possibly during school
3	Midday	14:00–14:30	Possibly during school
4	Afternoon	16:30–17:00	Prior to Dutch dinner time
5	Evening	19:00–19:30	Possibly during sports
6	Late evening	21:30–22:00	Prior to average bed time

^a^ Prompts were randomly sent through during this time window.

### The engagement strategy

**FIGURE 1:**
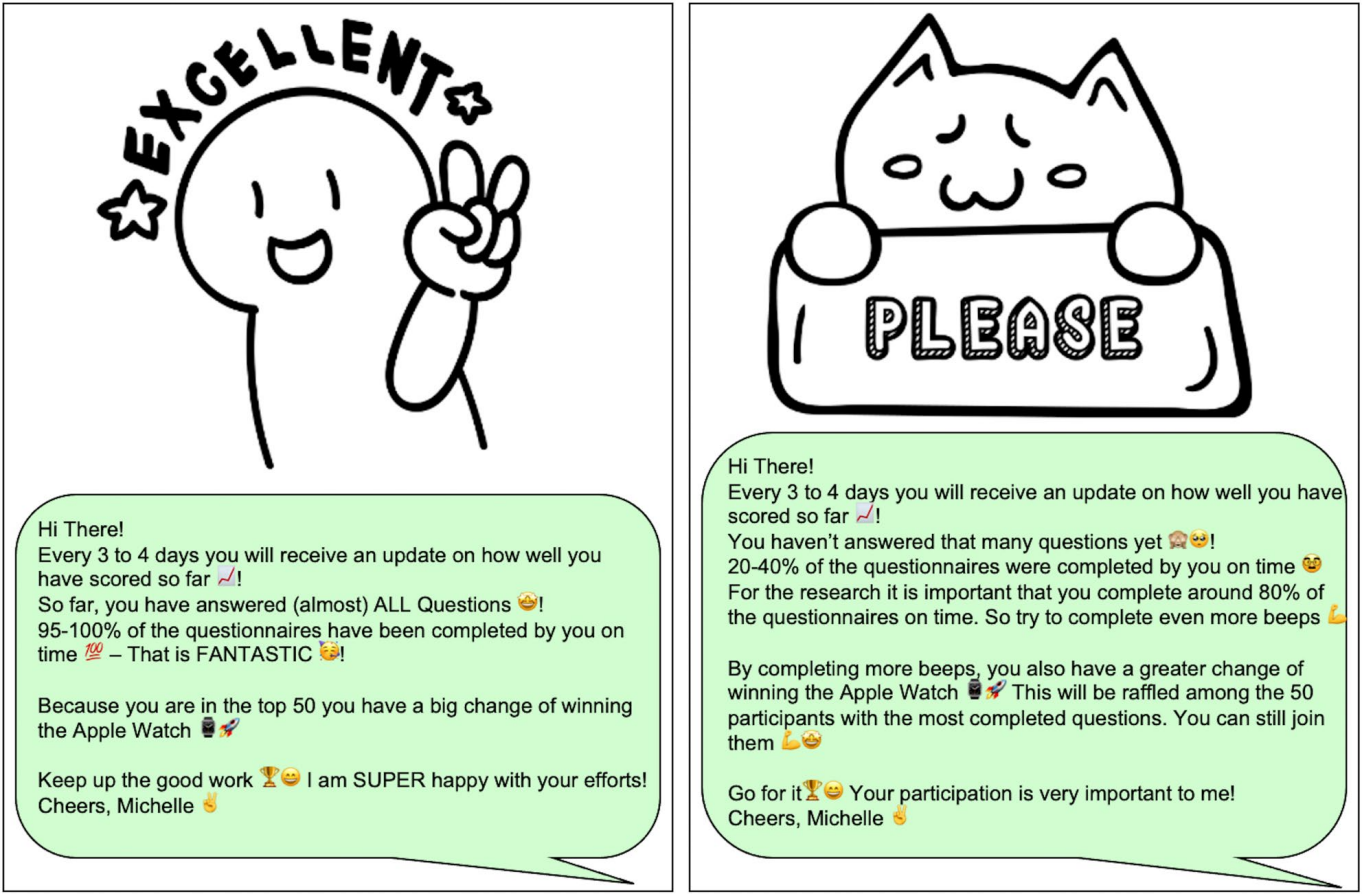
Examples of personalized compliance feedback through WhatsApp.

Given the constant stream of smartphone notifications adolescents receive (Radesky et al., 2023), a targeted engagement strategy was essential to support compliance during the 17‐day ESM protocol. To help the study remain visible and engaging amid adolescents' daily phone use, the ESM app (Avicenna) was paired with WhatsApp—a platform already embedded in their everyday routines. To foster a sense of belonging, share reminders, and maintain low‐threshold contact with participants, a WhatsApp Community was used—a broadcast that allowed one‐way group communication while preserving participant anonymity. All communication came from a single identifiable researcher (M.A.) to enhance trust and person‐related engagement (Bülow et al., 2025). On days 3, 6, 9, 13, and 16, short “Fool's Dilemmas” were included in Avicenna as additional questions, and group‐level results were shared in the WhatsApp Community, adding a playful social layer and reinforcing curiosity. This gamified element reflects key drivers of adolescent engagement—fun, autonomy, and relatedness (Bülow et al., 2025). Last, in addition to daily financial incentives, participants could win an Apple Watch via a prize lottery for the top 50 most compliant participants. Personalized feedback on compliance was sent via WhatsApp on days 4, 8, 11, and 15, based on Avicenna's real‐time progress tracker (Figure 1). All engagement materials are available via OSF metadata.

## RESULTS

### Feasibility under constraints: Compliance of the case study

To assess overall feasibility of the design, the percentage of completed prompts per day and per moment were calculated using R version 4.4.3 (R Core Team, 2023). A promp**t** was considered completed if the participant responded to at least one substantive item (e.g., *How irritated do you feel right now?*). On average, 78% of prompts were completed across participants (95% confidence interval [CI: 74.7%–80.6%], SD = 0.19, range = 18%–100%). This is comparable to general ESM compliance levels across samples (*M* = 79%; Wrzus & Neubauer, 2023), and slightly above the average reported for adolescent samples specifically (*M* = 74%; van Roekel et al., 2019). Interestingly, daily compliance was slightly higher on weekdays (80%) compared to weekend days (75%). These findings suggest that initial concerns regarding reduced compliance due to school smartphone bans and additional smartphone restrictions may be less pronounced than expected. However, such effects are likely conditional on the awareness and support of parents and school personnel, which may enhance adolescents' willingness to complete prompts and may, at least in part, reflect our deliberate efforts to engage parents and school administrators.

To explore individual differences in compliance, correlations and group comparisons were conducted. As part of the daily diary questionnaire (DDq, see OSF metadata), administered each morning through the Avicenna app, participants reported their total smartphone screen time for the previous day (hh:mm) and their screen time for the social category (e.g., WhatsApp, Instagram, Snapchat). For the present study, average values across the 17 study days were calculated for both total and social screen time to examine their associations with compliance. Compliance rates did not significantly correlate with age, total screen time, or social screen time (all *p* > .05). Gender differences were observed, with higher compliance among girls (*M* = 80.9%, SD = 16.3) compared to boys (*M* = 74.1%, SD = 20.9), *t*(174) = −2.52, *p* = .013, Cohen's *d* = 0.36, indicating a small‐to‐moderate effect. School type (VMBO, HAVO, VWO, or other) was not significantly associated with compliance, *F*(3, 189) = 1.29, *p* = .280, *η* ^2^ = 0.02.

**FIGURE 2:**
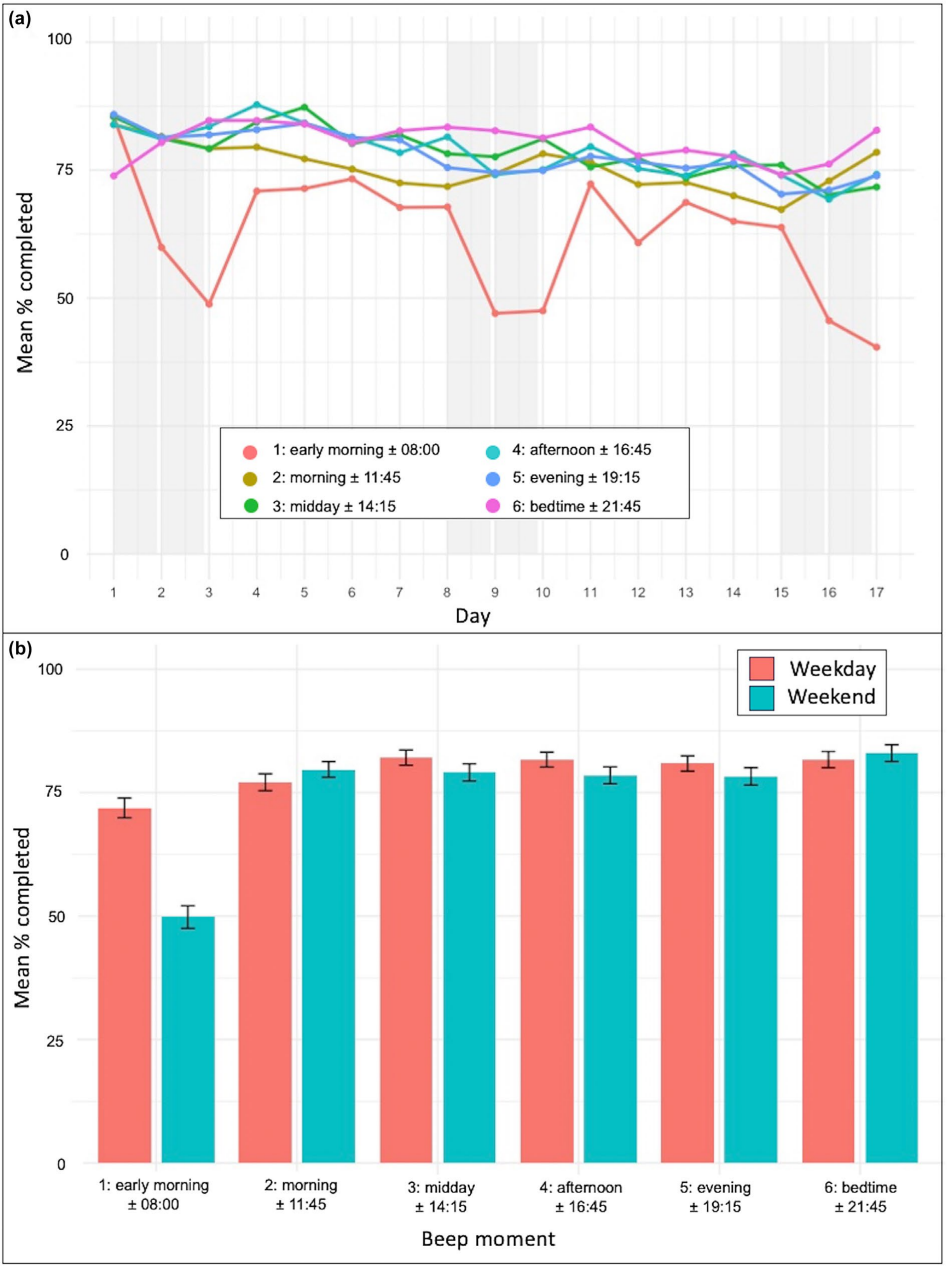
(a) Percentage completed prompts per day per moment across the 17‐day ESM study. Gray bars represent weekend days. (b) Compliance per moment for weekdays versus weekend. Error bars represent standard error of mean. ESM, experience sampling method.

As visualized in Figure 2a, clear patterns emerged across the day: Morning compliance was consistently lower compared to other moments, with especially reduced completion around 08:00 a.m. Additional comparisons examined completion rates per moment across weekdays and weekends (Figure 2b; Table 2). Compliance rates were examined for weekdays versus weekends across beep moments. The most pronounced effect was observed at the early morning prompt (±08:00), with substantially higher compliance on weekdays (*M* = 71.9%, 95% CI [68.0, 75.9]) than on weekends (*M* = 49.8%, 95% CI [45.3, 54.3]), *t*(195) = 11.40, *p* < .001, Cohen's *d* = 0.82, reflecting a large effect. Smaller differences were found in the afternoon (±16:45) and evening (±19:15), both favoring weekdays, although these did not survive Bonferroni correction (adjusted *α* = .0083). At the remaining prompts (morning ±11:45, midday ±14:15, and bedtime ±21:45), weekday–weekend differences were small and not statistically significant (all *p* > .050). Importantly, no substantial differences were observed during school hours (±11:45 and ±14:15) in weekdays versus weekend days. Across the 17‐day period, compliance slightly declined, potentially due to reduced motivation over time. This pattern supports previous findings that prolonged ESM protocols can increase perceived burden and reduce data quality (Eisele et al., 2022; van Roekel et al., 2019). Together, these results highlight that while compliance in adolescent ESM studies remains within an acceptable range, nuanced design choices and moment‐specific dynamics play a critical role in maintaining engagement.

**TABLE 2:** Mean compliance rates (%) for weekdays and weekends across measuring moments.

Beep moment	Weekday *M* [95% CI]	Weekend *M* [95% CI]	*t* (df)	*p*	Cohen's *d*
1. Early morning ±08:00	71.9% [68.0, 75.9]	49.8% [45.3, 54.3]	11.4 (195)	<.001*	0.82
2. Morning ±11:45	77.1% [73.7, 80.5]	79.7% [76.5, 82.8]	−1.56 (195)	.120	−0.11
3. Midday ±14:15	82.1% [79.1, 85.2]	79.1% [75.6, 82.5]	1.89 (195)	.060	0.14
4. Afternoon ±16:45	81.7% [78.7, 84.7]	78.5% [75.2, 81.9]	2.34 (195)	.020	0.17
5. Evening ±19:15	80.9% [77.9, 84.0]	78.3% [74.8, 81.7]	2.02 (195)	.044	0.14
6. Bedtime ±21:45	81.7% [78.4, 85.0]	83.0% [79.7, 86.4]	−0.98 (195)	.330	−0.07

Abbreviation: CI, confidence interval.

* Significant after Bonferroni correction (*α* = .0083).

### The missed moments: Item nonresponse

In the Avicenna app, participants can view their own progress: They can see which prompts they have completed and which ones were too late (expired). Each day, as part of the Daily Diary Questionnaire (DDq, see OSF metadata), we asked participants which prompts they did not complete the day before and for which reasons they did not complete specific prompts using a multiple choice question. This questionnaire was sent at 07:00 in the morning through the Avicenna app, and participants were able to complete the questionnaire until 23:59.

**FIGURE 3:**
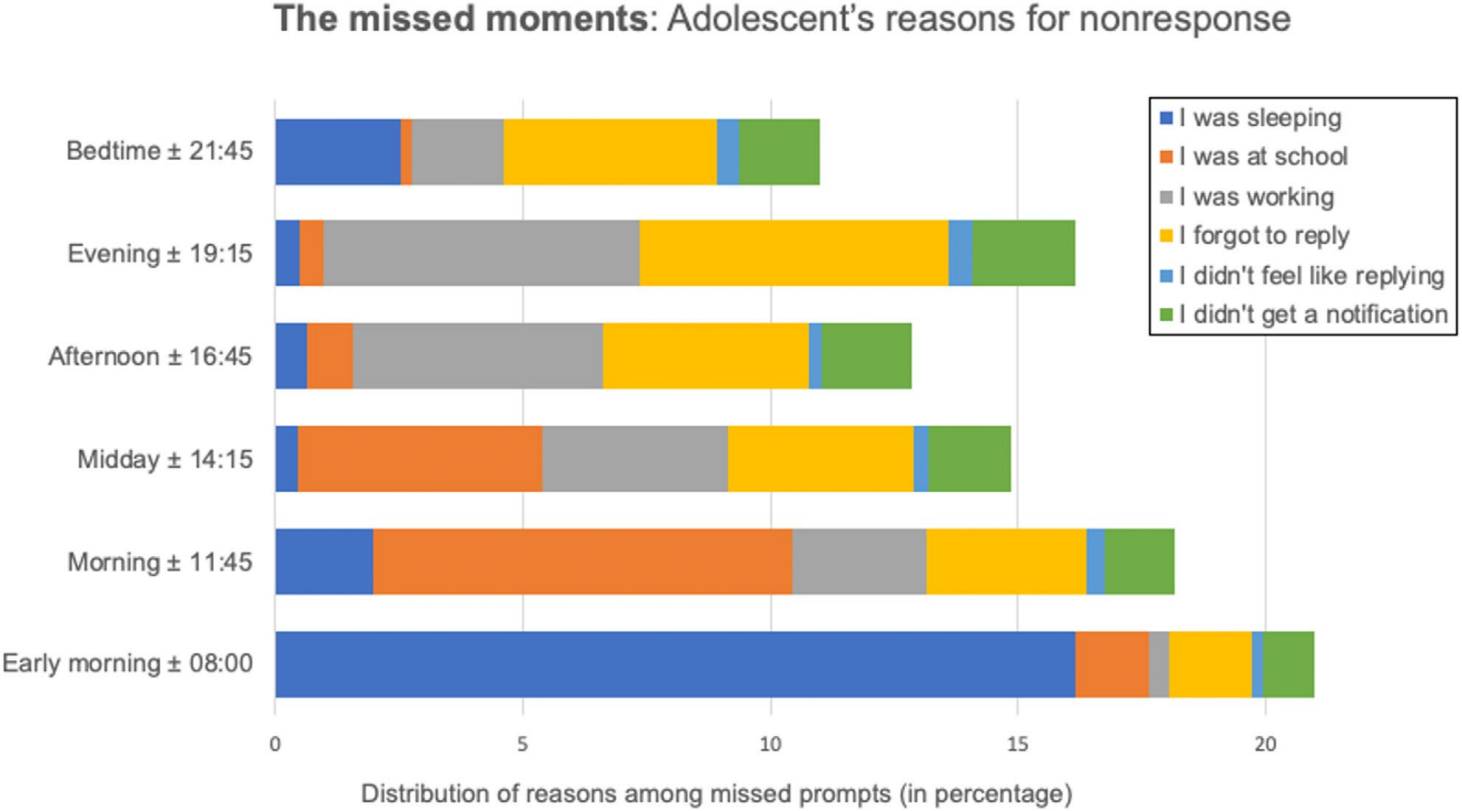
Adolescents' reasons for missing questionnaires per prompt.

As shown in Figure 3, the early morning prompt (±08:00) was most often missed because participants were still asleep, while the late morning prompt (±11:45) was primarily missed due to school‐related constraints. Based on these findings, it may be advisable to implement separate sampling schedules for weekdays and weekends (van Roekel et al., 2019). Evening prompts were often missed due to work—highlighting that nonresponse stems from both institutional and personal demands in adolescents' daily lives.

This interpretation aligns with recent conceptualizations of digital inaccessibility that extend beyond policy‐level constraints to include adolescents' self‐regulated unavailability due to competing priorities (e.g., extracurricular work, social obligations; see Vanden Abeele, 2020). It underscores the importance of assessing not just whether prompts are missed, but why—as such data reveal how availability fluctuates across the day and across individuals. Without this information, researchers risk misattributing missingness to lack of motivation or technical failure.

Figure 3 also shows that the reason “I forgot to reply” was reported across all time points, despite automated app reminders. This suggests that nonresponse may reflect more than just external constraints; it likely also stems from attentional demands and the cognitive burden of multitasking in notification‐saturated environments (Radesky et al., 2023). This highlights that adolescents' unavailability is shaped not only by school or parental policies, but also by internal rhythms, shifting priorities, and momentary disengagement (Vanden Abeele, 2020). Prompt‐level nonresponse data, as examined here, offer valuable insight into when and why adolescents fail to respond and can inform more ecologically grounded ESM designs. Importantly, smartphone constraints alone may not fully explain disengagement. By avoiding prompts during potentially disruptive school periods, researchers may inadvertently miss critical emotional fluctuations—especially those tied to academic or peer‐related stress (Silk et al., 2003). Rather than assuming that all missed responses are due to external restrictions, researchers should consider how cognitive load, timing, and competing demands shape adolescents' capacity to respond in real time. Although randomized, signal‐contingent prompting is often seen as the gold standard for assessing mood or emotions, participants may still delay or avoid responses during demanding moments (van Roekel et al., 2019). This raises the question of whether “in‐the‐moment” always equates to “most accurate” and highlights a broader tension in ESM methodology: balancing real‐time data collection with representative sampling (Myin‐Germeys et al., 2018). As others have noted (Bülow et al., 2025), overly rigid adherence to randomness may overlook the realities of adolescent routines and cognitive load.

### Parental and institutional restrictions on smartphone use: Qualitative insights

To get insight into possible smartphone constraints, participants were also asked about parental constraints or parent–child agreements on smartphone use in the Start Questionnaire (OSF metadata). Fifty‐six percent of the participants indicated that there were rules on when they were *not* allowed to use their smartphone. The most mentioned rules were “No phone during dinner” (48%), “No phone in bed in the evening” (25%), and “No phone during school hours” (20%). Despite rules on evening phone use, response rates at 21:45 remained high—suggesting timing and communication mitigated restrictions. This suggests that adolescents were generally able to respond to the bedtime prompt, despite parental restrictions. One possible explanation is that the prompt was scheduled early enough in the evening to avoid conflict with bedtime routines (Carskadon, 2011). Alternatively, the informational materials provided to parents and adolescents may have created space for flexibility—either by securing temporary exceptions to household rules or by legitimizing phone use as scientific. This observation highlights how timing and communication strategies can play a key role in navigating informal digital restrictions in ESM research with adolescents.

Additionally, smartphone restrictions during school hours were questioned using the School Smartphone Ban Questionnaire and the Exit Questionnaire (OSF metadata). Despite the fact that 88% of the participants had smartphone regulations at school (Brummer & Achterberg, 2025), participants reported considerable variation in the consequences of these restrictions: 48% were allowed to complete questionnaires at school, 25% were not, and 24% said it was sometimes permitted. Among those who were allowed, many obtained permission by explaining the scientific purpose—often supported by the study letter—while others noted lenient school policies or completed questionnaires during breaks. Those who were not allowed commonly cited strict school‐wide bans, teacher adherence to rules, or logistical issues such as exams or failing to seek approval in time. In some cases, students chose to comply with rules to avoid conflict, whereas others relied on completing the surveys using informal strategies, such as completing surveys during bathroom breaks or class transitions. Thus, contrary to the initial concerns, the majority of participants reported being able to complete ESM prompts at school. These findings echo our previous public report (Brummer & Achterberg, 2025) showing substantial variation in how smartphone policies are implemented in practice.

The present findings suggest that adolescents actively develop strategies to respond despite restrictions, and that the nationwide school ban did not result in lower compliance within this study. However, this raises important ethical considerations. Encouraging adolescents to respond during school hours—particularly when official policies prohibit smartphone use—may place an undue burden on them to navigate conflicting expectations. Some participants reported responding covertly during breaks, transitions, or even in bathrooms. While such efforts demonstrate their motivation and engagement, they also highlight the pressure adolescents may feel to accommodate research demands in restrictive environments. This dynamic warrants careful reflection for researchers: are we implicitly asking young people to circumvent rules or initiate conversations with authority figures that may make them uncomfortable? While scheduling prompts outside classroom hours diminishes these ethical constraints, it potentially compromises ecological validity (Scollon et al., 2003). By avoiding moments when participants are actively engaged in school activities, researchers risk missing not only key fluctuations in emotion and stress but also the very contexts in which these daily‐life experiences most often occur (Silk et al., 2003). Future ESM studies in similarly regulated contexts should weigh the benefits of in‐the‐moment data capture against the potential cost of placing adolescents in ethically ambiguous situations.

It is important to note that adolescents' actual availability during school‐time prompts may vary not only because of differences in smartphone policy implementation but also due to individual school schedules (e.g., free periods, early release, timetable variation). In addition, as described earlier in the manuscript, several adolescents indicated that they avoided responding during lessons to prevent conflict or disciplinary consequences (e.g., phone confiscation), suggesting that enforcement climate may also play a role in school‐time feasibility. Such structural and contextual differences in daily routines may obscure the extent to which institutional rules truly shape in‐the‐moment feasibility. Future research should therefore systematically capture both school‐level policy characteristics and students' timetable information to better disentangle the relative contribution of institutional restrictions versus individual availability in shaping school‐time compliance.

### Adolescent engagement and integrity

To evaluate both the quality and experience of adolescent participation, objective indicators of response validity were combined with subjective reports of user engagement. Objective indicators of response quality suggested overall high attentiveness. Following common practices in ESM research (e.g., Revol et al., 2024), responses under 3 s to three emotion‐related items (e.g., “How irritated/cheerful/stressed do you feel right now?”) were flagged as careless, yet such responses occurred in only 14.3% of participants. This is in line with prior findings suggesting that brief moments of disengagement are common, but typically non‐systematic (Meade & Craig, 2012; Revol et al., 2024). “Long string” responses—identical answers to all four items—were even rarer, comprising just 0.08% of all entries. Importantly, these responses were not excluded from the analyses, in order to avoid potential sampling bias, as adolescents experiencing greater burden may be more likely to provide such responses. Instead, the overall rate of potentially careless responding is reported here to provide transparency. The objective patterns were supported by participants' self‐reported honesty: on a scale of 0 to 100, participants rated their own honesty at 91 and estimated others' honesty at 80. Open‐ended responses confirmed these impressions, with most participants citing scientific relevance and anonymity as key reasons for answering truthfully.

Participant feedback from the exit questionnaire indicated that, although completing six prompts and one DDq per day was sometimes experienced as demanding, the overall burden was perceived as manageable. Importantly, 99% of adolescents indicated they would be willing to participate again in a similar study, suggesting that the combined use of Avicenna for prompts and WhatsApp for reminders was well received and did not result in excessive cognitive load. On average, participants rated their overall experience positively (*M* = 69.43 on a 0–100 scale) and gave the study a grade of 7.5 (out of 10). Most participants were motivated by multiple factors: 63% mentioned financial incentives, 41% were driven by the opportunity to contribute to science, and 30% were motivated to help the principal investigator. Interestingly, 58% reported participating annually as a motivating factor, suggesting a sense of continuity and investment in the project. Open‐ended feedback reinforced these motivations, with many describing the Apple Watch lottery or top 50 ranking as engaging. However, some participants noted areas for improvement: the 8:00 a.m. prompt was often considered too early—especially on weekends—and some found the item repetition monotonous. These comments point to the value of aligning design features more closely with adolescent rhythms and preferences.

Taken together, these findings suggest that adolescent ESM participation can be both feasible and reliable—when supported by thoughtful design choices. While careless responding was minimal and honesty self‐reports high, participant feedback underscores the importance of dynamic engagement strategies. Future studies should incorporate brief, low‐burden validity checks (e.g., fast responses, long strings) alongside exit‐based feedback tools to monitor and adapt study design.

## DISCUSSION

### Compliance, constraints, and context: The case study summarized

This study examined the feasibility of ESM research in adolescents under the growing constraints of smartphone use driven by shifting societal norms around digital hygiene and well‐being (i.e., school‐wide smartphone bans, parental regulations, and adolescents' own decisions to limit phone use). Through adaptive study design—scheduling prompts around school breaks, engaging participants via preferred communication tools (e.g., WhatsApp), and providing institutional letters—a moderate‐to‐high compliance (78%) was achieved with minimal signs of careless responding. Although the present study was not designed to examine gender or age differences in depth, our findings align with prior ESM work showing somewhat higher compliance among girls compared to boys (van Roekel et al., 2019; Wrzus & Neubauer, 2023). Such differences may reflect established gender patterns in conscientiousness (Schmitt et al., 2008; Weisberg et al., 2011) or willingness to participate in surveys (Singer et al., 2000), and underline the importance of balanced recruitment strategies in adolescent ESM research. Across the day, compliance was highest in the evenings and lowest in early mornings, consistent with adolescent sleep–wake rhythms (Chin et al., 2016). Importantly, no substantial drops were observed during school hours, despite national wide school smartphone bans. These findings suggest that, despite increasingly regulated digital environments, real‐time data collection remains feasible when protocols are thoughtfully aligned with both school structures and adolescent biological rhythms. In addition to accommodating external constraints, researchers should consider internal rhythms—such as later waking patterns on weekends—when designing ESM schedules.

Our results support Revol et al.'s (2024) recommendation to build design flexibility into ESM protocols to safeguard data quality in ecologically valid, real‐world settings. However, such adjustments highlight a fundamental tension in ESM research: the balance between ecological validity—capturing experiences in their natural context—and the need for standardization of research protocols to ensure comparability and rigor. In this study, design adaptations such as wider response windows and context‐sensitive scheduling were necessary to safeguard feasibility and participant engagement, yet they also introduced deviations from strict randomization. However, rather than viewing these adjustments as methodological compromises, they represent deliberate trade‐offs that maintain ecological validity while retaining sufficient standardization to ensure data quality and scientific value.

It should be noted that the sample was relatively homogeneous in terms of ethnic background, with 99% of participants identifying as ethnically Dutch, and a majority of families reporting higher parental education. This limits the generalizability of findings, particularly when considering contexts with more diverse populations, such as the United States, where adolescents are more heterogeneous in both ethnicity and socioeconomic background. Future studies should therefore aim to recruit more diverse samples in terms of ethnicity and socioeconomic background to explore whether compliance with ESM protocols differs across these dimensions. Moreover, as with many ESM studies, the possibility of reactivity effects such as the Hawthorne effect cannot be excluded. However, participants were not aware that compliance would be analyzed, reducing the likelihood of systematic bias in the present findings.

Despite these methodological considerations, the findings of the current study are highly valuable. This is the first study to examine adolescent ESM compliance in the context of nationwide smartphone bans. The results provide timely and important evidence that compliance remains feasible under restrictive conditions, particularly when adolescents, parents, and school staff are engaged in the process. As such, the study not only contributes to methodological discussions on balancing ecological validity and feasibility, but also provides a foundation for researchers internationally who aim to implement ESM in real‐world school settings.

This study contributes to the field by empirically illustrating the diversity of constraints adolescents face and by proposing adaptive strategies that maintain scientific integrity under real‐world limitations. Smartphone constraints—such as parental rules and school smartphone bans—should not be viewed merely as barriers to data collection, but as manifestations of broader societal negotiations around digital well‐being (Vanden Abeele, 2020). Table 3 presents a structured overview of these constraints, along with possible design solutions and their methodological trade‐offs. By adopting this nuanced, multidimensional approach, ESM research can continue to evolve in ways that are both ecologically grounded and methodologically sound.

**TABLE 3:** Navigating smartphone restrictions in adolescent ESM.

Challenge	Adaptive strategy	Methodological considerations
Smartphone bans during school classes	Schedule prompts during breaks or transitions from school to home	Risk missing classroom‐related emotional dynamics
Strict enforcement by teachers	Provide official study letter, inform staff, and highlight scientific relevance	May rely on institutional approval or collaborations; adolescents do not always inform teachers themselves
Parental bedtime phone bans	Send prompts just before bedtime	Possibly miss late‐evening affective fluctuations
Early morning sleep patterns	Avoid too early prompts on weekends	Increases feasibility but may reduce within‐day coverage
Cognitive overload from notifications	Use ESM app in flight mode to prevent notifications from other apps	Improves focus but excludes digital social context that may shape emotional dynamics
Adolescent disengagement	Use WhatsApp for reminders and gamification	May increase notification stream and cognitive overload

Abbreviation: ESM, experience sampling method.

### Sampling what matters: Implications for future adolescent ESM

Rather than advocating for a universal ESM protocol, this study highlights the need for context‐sensitive and question‐driven designs. Design choices—such as wider response windows and scheduling around school breaks—were guided by both practical constraints (e.g., smartphone bans) and the aim to capture adolescents' real‐world affective experiences (Silk et al., 2003). For research targeting in‐school stressors, avoiding class‐time prompts may compromise ecological validity. Feasibility, therefore, should be treated as multidimensional, encompassing data quality, participant experience, and alignment with study goals (Bülow et al., 2025; Revol et al., 2024). While random sampling may be crucial in some cases to test time‐sensitive hypotheses, modest deviations—like avoiding high‐disruption moments—can enhance engagement without sacrificing rigor. Our findings show that institutional rules do not always equate to reduced feasibility: many students still responded during school hours, enabled by school culture, teacher discretion, or informal strategies. However, structural differences in school‐level smartphone policy and access introduce the possibility that missing data during school hours are not missing at random, but systematically shaped by institutional constraints. This aligns with methodological work showing that ESM missingness is often contextually patterned (Eisele et al., 2022; Myin‐Germeys et al., 2018) and that adolescents' availability differs across structured daily contexts such as school versus home (van Roekel et al., 2019). Future research may therefore consider context‐sensitive adaptations to sampling schemes, as also recommended by ESM methodological guidelines (Myin‐Germeys & Kuppens, 2022), to ensure that participants facing the strictest restrictions are not structurally disadvantaged.

In practice, realizing a fit‐for‐purpose approach benefits from participatory co‐design. Therefore, a further consideration concerns the importance of co‐creation with key stakeholders—parents, school administrators, and adolescents themselves—when designing and implementing ESM studies, regardless of whether smartphone bans are in place. Collaborating with adults in adolescents' daily environments may strengthen recruitment, foster trust, and support adherence throughout the study period. These considerations also intersect with developmental differences in the degree of autonomy granted by parents, who remain important gatekeepers of device access throughout adolescence. At the same time, involving adolescents not only as participants in pilot testing but also as co‐creators in shaping study design decisions (i.e., Youth‐Participatory Action Research [YPAR]) can enhance feasibility and ecological validity (Anyon et al., 2018; Toenders et al., 2024). Such engagement may be especially relevant when considering sensitive questions or contexts, which might influence response timing (e.g., answering late at night) and perceptions of burden. Moreover, future research should examine whether compliance patterns differ when no incentives are provided or when restrictions and incentives interact with the type and sensitivity of questions being asked. Co‐creation with adolescents may help mitigate these challenges by aligning study demands with participants' daily realities and preferences (Bülow et al., 2025).

Beyond the specific context of smartphone bans, these findings also have broader implications for ESM studies of adolescent experiences in school settings, including research on environmental stressors such as discrimination or peer/parental conflicts. While “in the moment” reporting is often considered optimal for minimizing recall bias, there may be contexts where allowing delayed responding could reduce burden, protect privacy, or facilitate more thoughtful reflection (cf. Hektner et al., 2007; Myin‐Germeys et al., 2018). For instance, adolescents may feel more comfortable reporting sensitive experiences, such as discrimination or bullying, later in the day when they are in a safer environment. Future work should consider under which circumstances immediacy is essential and when flexibility may be more appropriate to balance ecological validity, feasibility, and ethical concerns.

## CONCLUSION

As societal norms around adolescent smartphone use continue to shift—particularly in educational contexts—adapting ESM designs has become essential to ensure both feasibility and scientific value. The case study demonstrates that high compliance and data quality can be achieved even under national school smartphone bans, provided that protocols are flexibly designed and attuned to the realities of adolescent life. While such adaptations inevitably introduce trade‐offs in terms of ecological validity and sampling precision, they also reflect a necessary evolution in methodological practice. By identifying concrete constraints and offering practical design strategies, this study contributes a novel, empirically grounded perspective on how to conduct rigorous, real‐time research with adolescents in increasingly restrictive digital environments. These findings hold particular relevance for the field of adolescent development, where capturing lived experience in context is vital—and where ESM, when carefully adapted, remains a powerful tool for this purpose.

## AUTHOR CONTRIBUTIONS

**Michelle Achterberg:** Conceptualization; formal analysis; funding acquisition; methodology and writing (original draft preparation and review and editing).

## FUNDING INFORMATION

This research was supported by a VENI grant awarded to Michelle Achterberg by the Dutch Research Council (NWO‐VENI grant number 17239).

## CONFLICT OF INTEREST STATEMENT

The author declares no conflicts of interest.

## ETHICAL APPROVAL STATEMENT

The procedures were approved by the Dutch Central Committee for Human Research (CCMO) on April 24, 2024 under reference number NL50277.058.14.

## PATIENT CONSENT STATEMENT

Informed consent was obtained from all participants, and if necessary (i.e., participants <16), from legal guardians.

## Data Availability

All materials related to this study, including questionnaires, study protocols, and communication materials, are publicly available via the Open Science Framework (OSF): https://osf.io/rb9e7/?view_only=b453394754cd4a5b80a60a03ec3b4b72. Due to ethical and privacy considerations, raw participant data are not publicly shared, but access to anonymized datasets may be granted upon reasonable request and with appropriate institutional approval.
